# Hepatoid adenocarcinoma in ureter with next-generation sequencing: A case report and literature review

**DOI:** 10.1186/s12920-023-01776-5

**Published:** 2024-01-02

**Authors:** Mengxin Lu, Yueying Li, Dongliang Hu, Jingtian Yu, Hang Zheng, Tongzu Liu

**Affiliations:** 1 Department of Urology, Zhongnan Hospital of Wuhan University, Wuhan, China; 2 Department of Pathology, Zhongnan Hospital of Wuhan University, Wuhan, China

**Keywords:** Ureter, Hepatoid adenocarcinoma, Chronic irritation, TP53 mutation

## Abstract

**Background:**

Hepatoid adenocarcinoma (HAC) is rare in the urinary system, with only 7 reported cases in upper urinary tract. This report aimed to explore the genetic characteristics of ureteral HAC for first time, and to describe the treatment prognosis of ureteral HAC.

**Case presentation:**

We present a rare case of ureteral HAC in a 53-year-old female, showing elevated serum levels of AFP and CEA, prolonged chronic irritation may be an important cause of her ureteral HAC. Radical nephroureterectomy was performed, the serum levels of AFP and CEA decreased significantly, and metastasis in lymph nodes was found at 9 months after surgery, she had no related symptoms after 18 months postoperatively without adjuvant chemotherapy. Three driver somatic mutations in cancer were identified by NGS testing, including: TP53^D281H^, KMT2D^L1211Ifs*2^, KMT2D^T1843Nfs*5^, demonstrating that ureteral HAC has the similar mutational features to upper tract urothelial carcinoma. Homologous-recombination deficiency (HRD) was positive in this tumor with no mutations in HRD-related genes, which was possibly induced by the copy number deletion of SETD2 gene.

**Conclusions:**

We report a rare case of ureteral HAC with elevated serum levels of AFP and CEA. NGS testing demonstrated that ureteral HAC has the similar mutational features to upper tract urothelial carcinoma, which is an important guide for the diagnosis and treatment of ureteral HAC.

## Background

Hepatoid adenocarcinoma (HAC) is a rare type of extrahepatic adenocarcinoma with production of alpha-fetoprotein (AFP), which has a morphological phenotype of hepatocellular carcinoma (HCC) [1]. In 1985, HAC was first described in a case of gastric adenocarcinoma by Ishikura et al. [2] Like the typical HCC, immunohistochemical (IHC) staining of HAC usually showed positive AFP stains (91.6%), together with elevated serum levels of AFP in most cases but not in all [1, 3]. Meanwhile, positive carcinoembryonic antigen (CEA) stains were also found in most HAC patients (78.7%) [1]. Until now, HAC has been reported in several organs, and the stomach is the most common location of HAC in the literature [1, 4].

HAC was rarely reported in the urothelium, as far as we know, only 17 previous HAC cases in the urothelium were reported in the literature, including 10 cases of bladder [4, 5], 7 cases of renal pelvis and ureter [6]. In the current report, we present a case of HAC located in the ureter with production of AFP and CEA. Moreover, next-generation sequencing was applied to explore its molecular characterization, this seems to be the first documentation to study the molecular characteristics of HAC in the urothelium.

## Case presentation

### Clinical findings

**Fig. 1:**
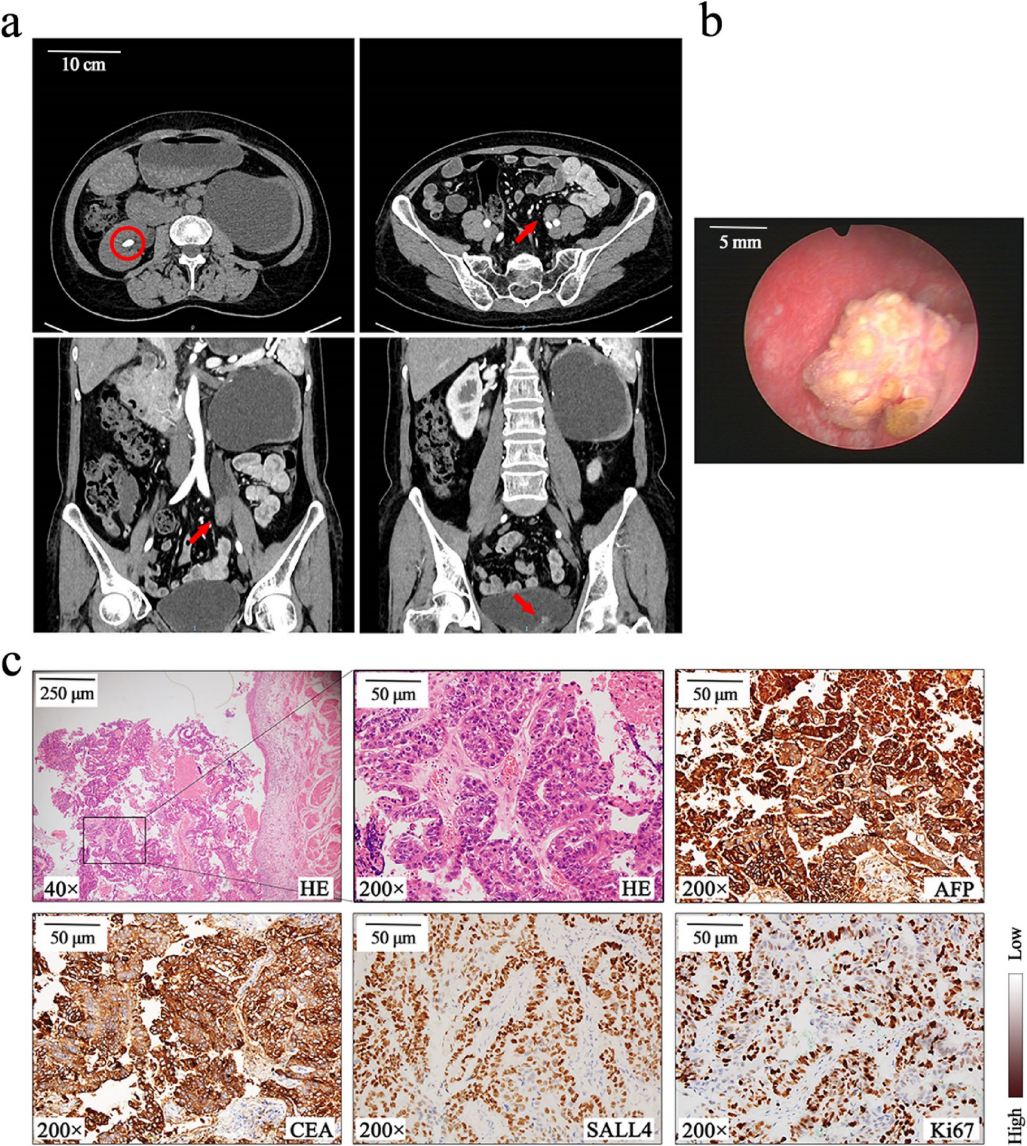
**a** CTU scan showed a stone in the right kidney (circle) and enhanced lesions in the middle and lower left ureter (arrow); **b** Cystoscopy revealed a solitary tumor on the left ureteral opening of the bladder; **c** Hematoxylin-eosin (HE) stain showed the transitional area between tumor and uroepithelium, and the tumor cells were distributed in a trabecular and glandular pattern. Tumor cells stained positively for AFP, CEA, SALL4 and Ki67

A 53-year-old Chinese female was admitted to our hospital for complaining of 2-weeks gross hematuria in May, 2021. Pulmonary CT (computed tomography) scan and computed tomography urography (CTU) scan (Fig. 1A) were applied, revealing multiple mass lesions with enhancement in the middle and lower left ureter, which are considered to be neoplastic lesions. In addition, CTU also showed a stone with a diameter of 2 cm in the right kidney, while there were no other primary or metastatic lesions, especially in the liver, lung and gut. Her history was significant for left ureteroscopic lithotripsy (URSL) and ureteral stent placement for left ureteral stone at another hospital in 2017. In the following years, she underwent multiple ureteral stent placements, due to the recurrent ureteral strictures. Moreover, in August 2018, she underwent total hysterectomy for cervical high-grade squamous intraepithelial lesions. She has a history of hepatitis B and has no history of smoking.

As part of her clinical workup, the fluorescence in situ hybridization result of her urine specimen was positive. Furtherly, cystoscopy was applied, revealing a solitary tumor on the left ureteral opening of the bladder (Fig. 1B). And the glomerular filtration rate (GFR) measured by 99 m Tc-DTPA renal dynamic imaging showed severe impairment of the left GFR (left GFR = 6.64 ml/min, right GFR = 54.65 ml/min). Serum AFP was detected to be 568.9 ng/ml (normal range < 6.6 ng/ml), serum CEA was 20.0 ng/ml (normal range < 7.2 ng/ml) and cancer antigen 199 (CA-199) was 99.51 U/ml (normal range < 37 U/ml). And serological markers for hepatitis B viruses HBV were positive (HBsAg+, HBeAb+, HBcAb+).

Based on these findings, the patient was diagnosed as having a tumor in left ureter combined with severe hydronephrosis of the left kidney, therefore radical nephroureterectomy was performed in May, 2021, with pathologically confirmation of HAC. Meanwhile, she underwent right ureteral stent placement combined with secondary retrograde intrarenal surgery for right kidney stone.

### Pathology and immunohistochemistry

Surgically removed specimens were fixed in 10% buffered formalin, hematoxylin-eosin (HE) stain showed a T3-stage tumor with invasion to the ureteral muscle layer. The tumor cells were distributed in a trabecular and glandular pattern with no conventional urothelial carcinoma, and the transitional area between tumor and uroepithelium was visible (Fig. 1C). Adjuvant chemotherapy (Gemcitabine-cisplatin, GC) is recommended because the tumor is T3-stage, but the patient refused it.

As shown in Fig. 1C, IHC stains demonstrated that the cancer cells were positive for anti-AFP, anti-CEA, anti-SALL4 (spalt like transcription factor 4). Moreover, 60% of Ki67 positivity indicated a higher proliferation rate in the tumor. Combining tumor morphology and IHC characteristics, the tumor was diagnosed as a HAC with ureteral origin after a multidisciplinary discussion [1, 7].

### Sequencing analysis

To explore the genomic alterations of ureteral HAC, next-generation sequencing (NGS) was performed to detected the mutational signatures in 537 cancer-associated genes. The NGS tests were done by Beijing Genomics Institute (Beijing, China). Firstly, germline variants testing using blood specimen revealed three mutations of unknown clinical significance, including: BRCA1^N909I^, IRS1^G596R^ and ALK^R672S^, and the detailed mutation information is shown in Table 1. According to the data provided in the ClinVar databases (https://www.ncbi.nlm.nih.gov/clinvar/), BRCA1^N909I^ is classified as likely benign, IRS1^G596R^ have not been reported, while ALK^R672S^ is classified as a variant of uncertain significance.

**Table 1:** Genomic alterations of ureteral HAC by NGS testing

Gene	Classification of mutation	Variation Location	Protein change	Interpretation	Gene function
BRCA1	Germline	g.17:41244822 NM_007294 c.2726A > T	N909I	Uncertain significance, likely benign.	Tumor suppressor, involved in DNA repair of double-stranded breaks, mutations in inherited breast and ovarian cancers.
IRS1	Germline	g.2:227661669 NM_005544.2 c.1786G > A	G596R	Uncertain significance	Mutations in this gene are associated with type II diabetes and susceptibility to insulin resistance.
ALK	Germline	g.2:29497990 NM_004304.4 c.2016A > T	R672S	Uncertain significance	A receptor tyrosine kinase, rearranged, mutated, or amplified in a series of tumors.
TP53	Somatic	g.17:7577097 NM_000546 c.841G > C	D281H	Likely pathogenic	Tumor suppressor, containing transcriptional activation, DNA binding, and oligomerization domains. Mutations in a variety of human cancers.
KMT2D	Somatic	g.12:49443740 NM_003482.3 c.3629_3630dup	L1211Ifs*2	Pathogenic	A histone methyltransferase that methylates the Lys-4 position of histone H3. Mutations in Kabuki syndrome and upper urinary tract urothelial cancer.
KMT2D	Somatic	g.12:49437151 NM_003482 c.5527dup	T1843Nfs*5	Pathogenic	

>: substitution; dup: duplication; fs: frame shift

Furthermore, formalin-fixed paraffin-embedded (FFPE) tumor tissue was used to detected somatic variants. Except for the same germline variants as the blood specimen testing, three driver somatic mutations in cancer were identified, including: TP53^D281H^, KMT2D^L1211Ifs*2^, KMT2D^T1843Nfs*5^ (Table 1). TP53^D281H^ is classified as a likely pathogenic mutation in ClinVar databases, which disrupts the p.Asp281 amino acid residue in TP53. While KMTD2 gene alteration is one of the most common mutations in upper tract urothelial carcinoma (UTUC) [8]. Additionally, the results of somatic copy number alterations testing showed copy number deletion of SETD2 gene and copy number amplification of AKT2 gene. And homologous-recombination deficiency (HRD) was positive in the tumor with an HRD score of 51 (HRD score of ≥42 on the myChoice HRD Plus assay), indicating that this patient may benefit from poly adenosine diphosphate–ribose polymerase inhibitors and platinum-containing therapy [9]. Moreover, the sequencing results revealed a low tumor mutational burden and microsatellite stability in this tumor, indicating a low sensitivity to programmed cell death-1 (PD-1) blockade for this patient [10].

### Follow-up

**Fig. 2:**
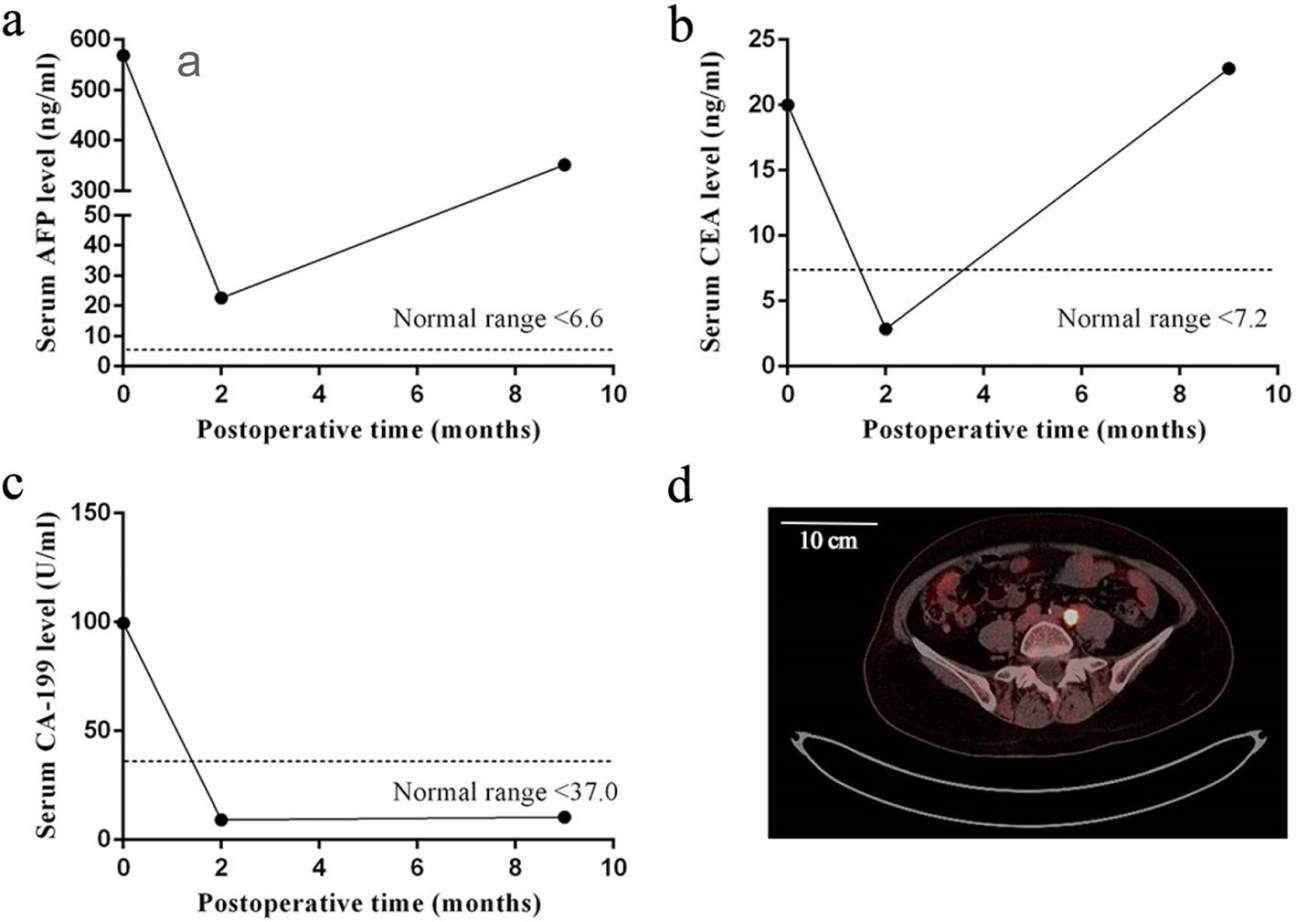
The follow-up of the serum levels of AFP **a**, CEA **b** and CA-199 **c**; **d**. ^18^F-FDG PET/CT scan showed metastasis in left common iliac lymph node after 9 months postoperatively

As shown in Fig. 2, after 2 months postoperatively, the serum levels of AFP, CEA and CA-199 decreased significantly, the serum AFP level was 22.7 ng/ml, CEA 2.87 ng/ml, CA-199 9.14 U/ml. At about 9 months after surgery, the patient returned to the hospital for a follow-up examination, and serum AFP increased to 351.7 ng/ml, CEA 22.8 ng/ml. Moreover, as shown in Fig. 2D, metastasis in retroperitoneal lymph nodes and left common iliac lymph nodes was found on ^18^F-fluorodeoxyglucose (FDG) positron emission tomography/computed tomography (PET/CT). GC chemical therapy was required, but the patient still refused it. After 18 months postoperatively, the patient had not come back for the second follow-up visit, and had no related symptoms when asked by telephone.

## Discussion and conclusions

HAC is a rare tumor with production of AFP, especially in the ureter and renal pelvis, a total of 8 cases of renal pelvis and ureter have been reported so far (Table 2). Except for case 3 whose AFP IHC information was not available, IHC staining were positive for AFP in all cases. Similarly, serum AFP levels increased in each case when available. Longstanding chronic inflammation and irritation due to calculi can induce primary adenocarcinoma in upper urinary tract [11–13]. Significantly, case 4 and two patients younger than 60 years (case 7 and our case) had the history of chronic irritation [6, 14]. Case 4 had an indwelling nephrostomy tube in the right kidney for many years, case 7 had the history of calculus in the right kidney, while our patient had undergone multiple ureteral stent placements for the recurrent ureteral strictures. Accordingly, we speculate that chronic irritation may be an important cause of ureteral and renal pelvic HAC, especially in the younger patients.

**Table 2:** Cases of ureteral and renal pelvic HAC in reviewed literatures

Case (Reference)	Nationality, year	Age, sex	History of chronic irritation	IHC AFP	Initial AFP (ng/mL)	Initial CEA (ng/mL)	Stage	Treatment	Post treatment AFP (ng/mL)	Outcomes
1 [15]	Japanese (1991)	72, Male	No	+	2246	NA	pT1	Surgery	2.6 (4 months after surgery)	DFS for 10 months, LFU
2 [16]	Japanese (1992)	68, Female	No	+	1220	NA	NA	Surgery	5.0 (2 months after surgery)	DFS for 12 months, LFU
3 [17]	Japanese (2002)	69, Female	No	NA	NA	24.5	pT2	Surgery	NA	DFS for 12 months, LFU
4 [14]	Japanese (2004)	70, Female	Yes	+	24,000	NA	pT2	Surgery and radiotherapy	Normal (4 months after surgery), increased after 9 months	LNM after 9 months, Dead after 28 months
5 [18]	Italian (2011)	80, Female	No	+	NA	NA	pT4	Surgery	NA	NA
6 [19]	Australian (2012)	84, Female	No	+	701	4.2	pT3	Surgery	Normal (5 months after surgery)	DFS for 5 months, LFU
7 [6]	Chinese (2019)	59, Male	Yes	+	64	NA	NA	Surgery	2.5 (1.5 months after surgery)	DFS for 3 months, LFU
8 (our case)	Chinese (2022)	53, Female	Yes	+	569	20.0	pT3	Surgery	22.7 (2 months after surgery)	LNM after 9 months, AWD for 18 months

IHC: immunohistochemical; NA: not available; DFS: disease-free survival; LFU: lost to follow-up; LNM: Lymph node metastasis; AWD: alive with disease

All cases of ureteral and renal pelvic HAC in the literature were treated with radical nephroureterectomy, and serum AFP decreased to normal level in 86% (6/7) of patients within 1.5–5 months postoperatively (case 3 and case 5 are not available). Case 4 received radiotherapy because the metastasis in a retroperitoneal node was found after 9 months postoperatively, and the lymph node decreased in size by 80% after radiotherapy, demonstrating that ureteral and renal pelvic HAC is sensitive to radiotherapy [14]. None of the reported ureteral and renal pelvic HAC cases received chemotherapy, GC chemotherapy had been recommended to our patient for several times, but the patient refused it. The NGS testing of this patient proved the similar mutational features of ureteral HAC to UTUC, therefore, we conclude that GC chemotherapy is effective for ureteral and renal pelvic HAC, this need to be confirmed by further studies.

According to the case reports in the literature, the prognosis of HAC patients was poor in general, lymph nodes metastasis was found in 57.5% patients when HAC was diagnosed, and the median survival of all patients was 12 months [1]. However, the prognosis of HAC in the urothelium seems to be not very bad, which is manifested as no one died of the cancer within 1 year in the 4 cases followed for more than 12 months.

NGS testing was performed in this case to explore the genomic alterations of ureteral HAC, and BRCA1^N909I^ mutation was found. BRCA1^N909I^ is located in DNA binding domain of BRCA1, and classified as likely benign based on a multifactorial analysis [20]. Somatic variants detected by FFPE tumor tissue showed mutations in TP53 and KMT2D genes, demonstrating that ureteral HAC has the similar mutational features to UTUC [8]. Consistent with this, it was reported that gastric HAC had a similar mutation spectrum to gastric cancer but not HCC [21].

HRD results in impaired double strand break repair and is frequently observed in breast, ovarian and prostate cancers [22]. HRD was positive in this tumor with no mutations in HRD-related genes. However, the copy number deletion of SETD2 gene was observed, SETD2 (SET Domain Containing 2, Histone Lysine Methyltransferase) was reported to be required in homologous recombination repair by promoting recruitment of RAD51 [23], the copy number deletion of SETD2 gene may be the reason for why the ureteral HAC show HRD-positive with no mutations in HRD-related genes.

In conclusion, we present a rare case of ureteral HAC showing elevated serum levels of AFP and CEA, prolonged chronic irritation may be the main cause of her ureteral HAC. NGS testing was applied to explore the genetic characteristics of ureteral HAC for first time, indicating the driver somatic mutations in TP53 and KMT2D genes, and HRD positivity possibly induced by the copy number deletion of SETD2 gene, which need to be confirmed by further studies. Our fndings could be helpful for the clinical diagnosis and treatment of ureteral HAC.

## Data Availability

The raw sequence data reported in this paper have been deposited in the Genome Sequence Archive in National Genomics Data Center, China National Center for Bioinformation / Beijing Institute of Genomics, Chinese Academy of Sciences (GSA-Human: HRA005075) that are publicly accessible at https://ngdc.cncb.ac.cn/gsa-human [24, 25].

## Abbreviations

HAC: Hepatoid adenocarcinoma
HRD: Homologous-recombination deficiency
AFP: alpha-fetoprotein
HCC: hepatocellular carcinoma
IHC: immunohistochemical
CEA: carcinoembryonic antigen
CTU: Computed tomography urography
URSL: ureteroscopic lithotripsy
GFR: glomerular filtration rate
CA-199: cancer antigen 199
HE: hematoxylin-eosin
GC: Gemcitabine-cisplatin
SALL4: spalt like transcription factor 4
NGS: next-generation sequencing
FFPE: formalin-fixed paraffin-embedded
UTUC: upper tract urothelial carcinoma
PD-1: programmed cell death-1
